# Does Economic Policy Uncertainty Matter for Healthcare Expenditure in China? A Spatial Econometric Analysis

**DOI:** 10.3389/fpubh.2021.673778

**Published:** 2021-05-04

**Authors:** Pu Bai, Yixuan Tang, Weike Zhang, Ming Zeng

**Affiliations:** ^1^Carey Business School, Johns Hopkins University, Baltimore, MD, United States; ^2^School of Public Administration, Sichuan University, Chengdu, China

**Keywords:** economic policy uncertainty, healthcare expenditures, spatial spillover effects, spatial Durbin model, regional heterogeneity

## Abstract

A growing body of research has documented the determinants of healthcare expenditure, but no known empirical research has focused on investigating the spatial effects between economic policy uncertainty (EPU) and healthcare expenditure. This study aims to explore the spatial effects of EPU on healthcare expenditure using the panel data of 29 regions in China from 2007 to 2017. Our findings show that healthcare expenditure in China has the characteristics of spatial clustering and spatial spillover effects. Our study also shows that EPU has positive spatial spillover effects on healthcare expenditure in China; that is, EPU affects not only local healthcare expenditure but also that in other geographically close or economically connected regions. Our study further indicates that the spatial spillover effects of EPU on healthcare expenditure only exist in the eastern area. The findings of this research provide some key implications for policymakers in emerging markets.

## Introduction

**Figure 1:**
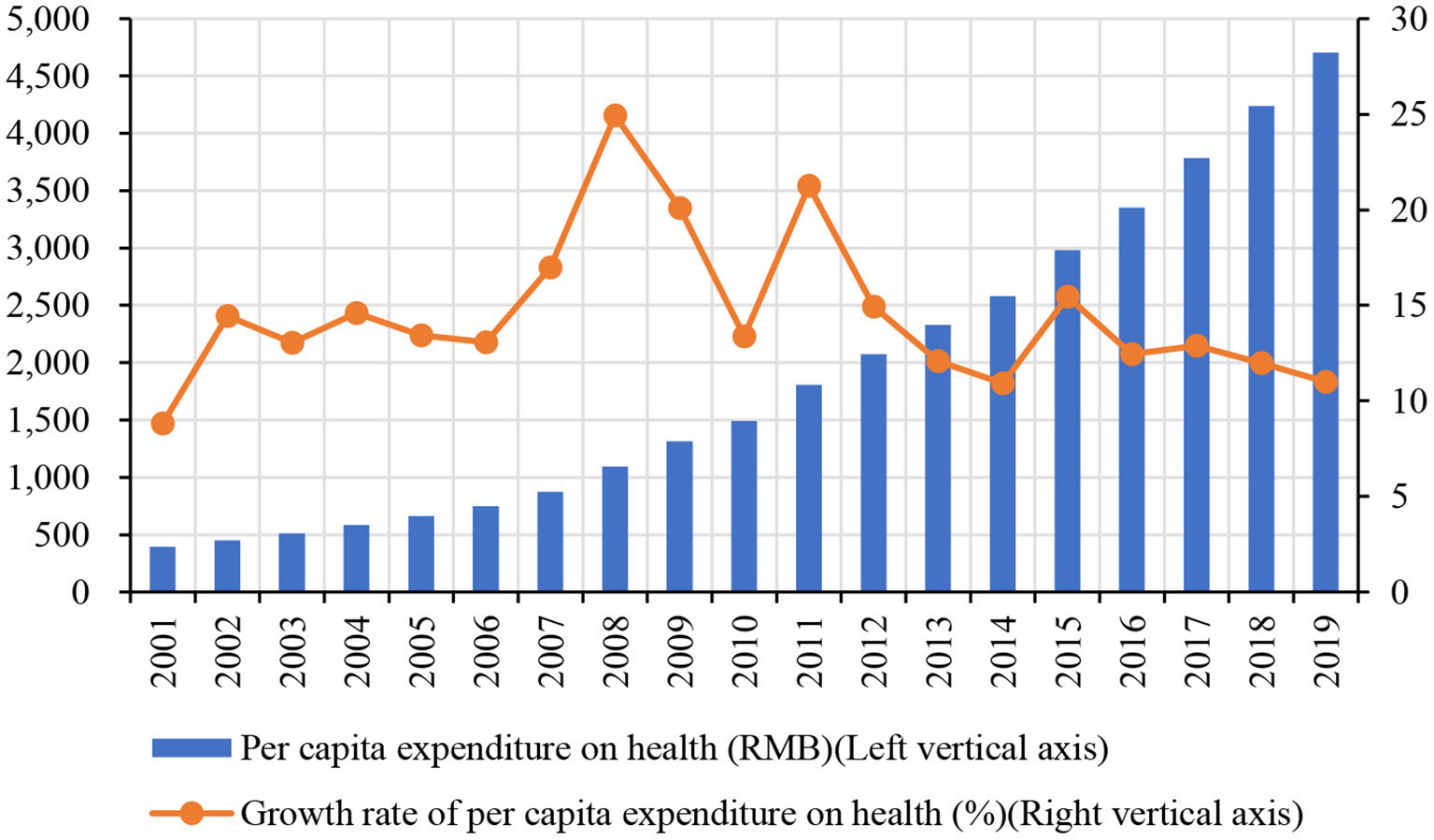
The trend of per capita expenditure on health in China (2000–2019). Data source: National Bureau of Statistics of China (https://data.stats.gov.cn).

Healthcare expenditure has increased rapidly with the development of China's economy in the past few decades. According to the National Bureau of Statistics of China, the ratio of total health expenditures to gross domestic product (GDP) increases from 4.57% in 2008 to 6.67% in 2019^1^. As shown in Figure 1, the per capita expenditures on health rose from 393.80 RMB in 2001 to 4,702.79 RMB in 2019, suggesting that the Chinese government increased funding to construct a more sustainable system that covers the health needs of most or all citizens. However, the growth rate of per capita expenditure on health fluctuates from 8.82% in 2000 to 10.99% in 2019, and it reaches a peak of 24.94% during the global financial crisis in 2008. These results indicate that health expenditure changes with economic growth and policies (1, 2).

In recent years, a large and growing body of literature has investigated the determinants of healthcare expenditure, such as business cycles (2), economic overheating (3), economic activity (4), economic crisis (1, 5), and other factors (6–10). The most studied determinant is economic growth, which is an important demand-side driver of healthcare expenditure (2, 11, 12). Some studies show that healthcare expenditure is significantly positive with GDP because the demand for healthcare services will rise with the increase of income (4, 11, 13). Furthermore, some studies find that other macroeconomic factors can also affect healthcare expenditure. For example, You and Okunade (14) state that technological changes and income are the major determinants of healthcare expenditure. Hyun et al. (15) find that population aging has a significant impact on healthcare expenditure but the conclusions are mixed.

In addition to the factors above, government policies have a key role in determining healthcare expenditure (11). Some studies show that governments can influence healthcare expenditure by formulating health policies to meet their objectives (16–18). For example, Cheng and Witvorapong (11) find that health policy uncertainty exerts a negative impact on health expenditures. Potrafke (19) investigates the impact of electoral motives on public healthcare expenditure using the data of 18 OECD countries and shows that the public healthcare expenditure increase significantly before election year for political opportunism of the incumbent government. These studies suggest that governments' health policies can affect healthcare expenditure, but the policies are uncertain and may be unpredictable at all. Economic policy uncertainty (henceforth EPU) represents the uncertainty of macroeconomic policies (20). The rise or fall of EPU has an impact on the economy and may prompt demanders and suppliers in the healthcare services market to respond in ways that policymakers cannot foresee (11, 21–23). Up to now, however, far too little attention has been paid to investigate the impact of EPU on healthcare expenditure in emerging market countries.

This study, therefore, set out to assess the spatial spillover effects of EPU on healthcare expenditure in China. The main contributions of this study are presented in two aspects. First, this study sheds new light on the impact of EPU on healthcare expenditure in China. To the best of our knowledge, only Cheng and Witvorapong (11) examine the link between health economic policy uncertainty and healthcare expenditure in the United States. However, unlike developed economies in Europe and the United States, China's health system is dominated by the government, which has great power to allocate resources directly (23–25). Hence, this study makes a major contribution to research on health economics by demonstrating the relationship between EPU and healthcare expenditure in emerging markets. Second, the importance and originality of this study are that it explores the spatial spillover effects of EPU on healthcare expenditure and their regional heterogeneity. To date, previous studies have failed to examine the spatial spillover effects between them. However, different regions are not independent of each other, and various economic activities in one region may affect economic activities in other regions (26, 27). If we ignore the spatial effects when analyzing the link between EPU and healthcare expenditure, the conclusion may be inaccurate. Therefore, we offer new empirical evidence on whether EPU has spatial spillover effects on healthcare expenditure in China. Our findings have some key implications for policymakers in emerging markets.

The remaining part of this study proceeds as follows: Section Data, variables and methods develops our research design, including data, variables, and methods. Section Spatial autocorrelation analysis presents the results of spatial autocorrelation analysis. Section Empirical analysis and discussion gives our empirical analysis and discussions. The final section concludes our research results.

## Data, Variables, and Methods

### Data

This study selects a sample of 29 regions in China from 2007 to 2017 to examine the spatial effects of EPU on healthcare expenditure. The samples include Beijing, Tianjin, Hebei, Shanxi, Inner Mongolia, Liaoning, Jilin, Heilongjiang, Shanghai, Jiangsu, Zhejiang, Anhui, Fujian, Jiangxi, Shandong, Henan, Hubei, Hunan, Guangdong, Guangxi, Chongqing, Sichuan, Guizhou, Yunnan, Shaanxi, Gansu, Qinghai, Ningxia, and Xinjiang. The data of EPU are selected from the EPU index proposed by Yu et al. (28). The data of inpatient expenditure and outpatient expenditure are collected from the China Health Statistics Yearbook 2008–2018. The other data are collected from the Chinese Statistics Yearbook 2008–2018.

### Variables

#### Dependent Variable: Healthcare Expenditure

Healthcare expenditure is one of the important indicators of residents' health status. Healthcare expenditure is used to measure the final consumption of healthcare goods and services, including personal healthcare (i.e., therapeutic care, rehabilitation care, long-term care, ancillary services, and medical goods) and collective services (i.e., prevention and public health services). In this study, following the studies of Pu et al. (2) and Zeng et al. (27), we use the inpatient expenditure (*in_exp*) as the proxy variable of healthcare expenditure, which refers to the logarithm of the per capita expenditure in hospitalization. Also, we use outpatient expenditure (*out_exp*) for the robustness test, which is expressed as the logarithm of the per capita expenditure in outpatient (27).

#### Independent Variable: EPU

**Figure 2:**
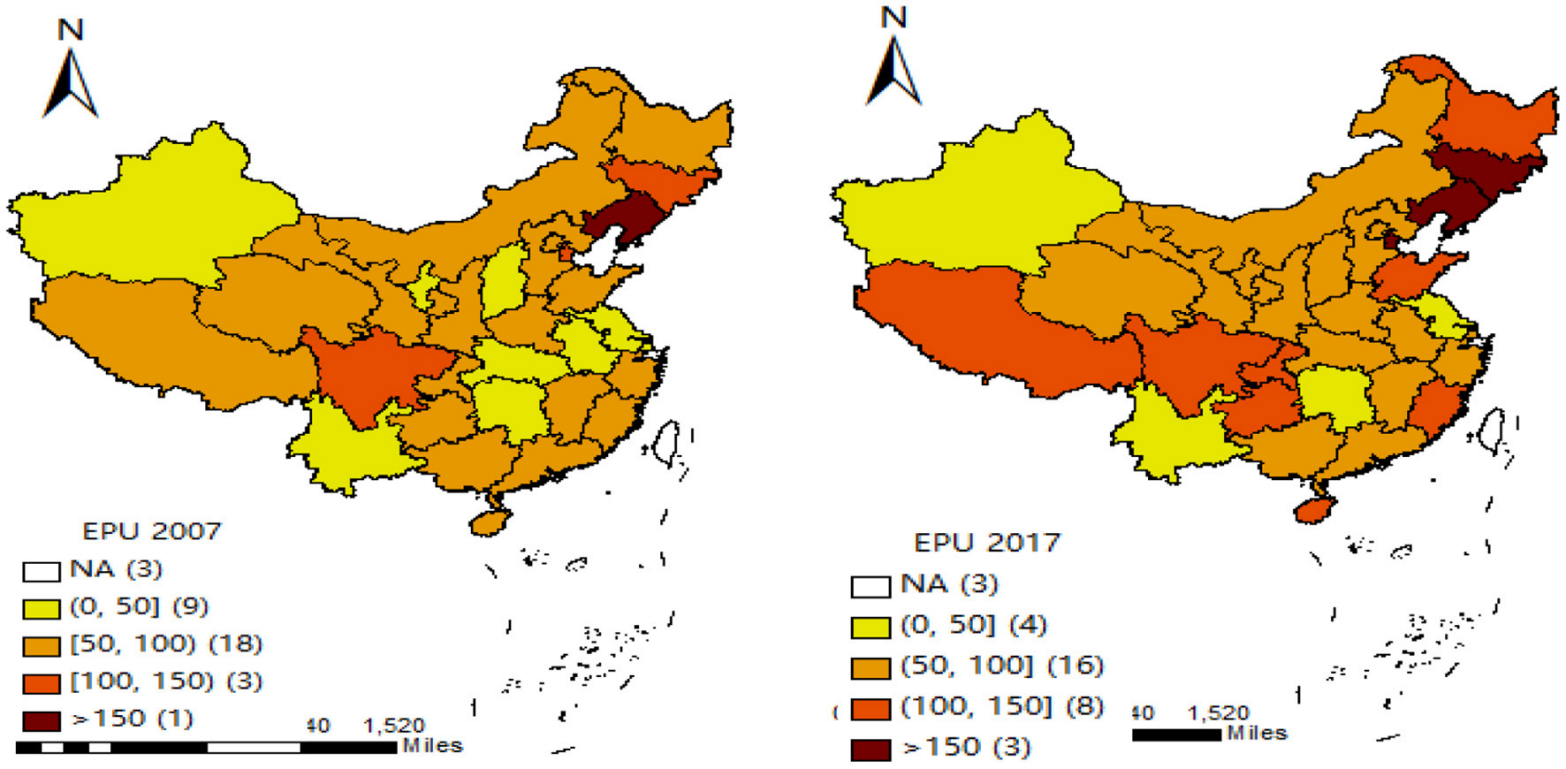
Distribution of the provincial EPU index in China (2007 and 2017).

The prior studies mainly use many proxy variables to measure EPU. For example, the single economic policy variables (29–32), the non-economic dummy variables (i.e., terrorist attacks and political events) (33–35), the EPU index proposed by Baker et al. (20) (BBI index) (23, 36–38). Since the measures above on EPU are mainly at the national level, the EPU of different regions in China may be different. Hence, we measure the provincial EPU (*EPU*1) using the EPU index proposed by Yu et al. (28). Besides, we use the standardization of China's provincial EPU index (*EPU*2) to conduct a robustness test. Figure 2 gives the distribution of China's provincial EPU index in 2007 and 2017. They show that the EPU index of different regions in China has obvious dynamic change characteristics and significant differences. For example, in 2007, the EPU index of Liaoning province is the highest, and that of Jiangsu province is the lowest. However, in 2017, the region with the highest EPU index is Shanxi province, and that with the lowest is Heilongjiang province.

#### Control Variables

According to the determinants published by the World Health Organization (WHO), many factors would affect healthcare expenditure. Following the study of Zeng et al. (27), we control the following variables: economic development (*GDP*), aging rate (*Aging_rate*), urbanization level (*Urban_rate*), industrial structure (*Indu_rate*), the mortality rate (*Mor_rate*), the number of medical institutions (*Hos_num*), fiscal revenue (*Fiscal_rev*), and the number of per capita beds in medical institutions (*Bed_num*). The economic development is measured by the logarithm of gross domestic product (GDP). The aging rate is measured by the ratio of people over 65 to the total population. The urbanization level is measured by the ratio of the urban population to the total population. The industrial structure is measured by the proportion of the third industry in the economic structure. The mortality rate is measured by the ratio of the deaths to the total population. The number of medical institutions is measured by the logarithm of the total number of medical institutions. The fiscal revenue is measured by the logarithm of the total number of government fiscal revenue. The number of beds in medical institutions is measured by the logarithm of the total number of beds in medical institutions per 10,000 people.

All variables and their definitions are given in Table 1, and their descriptive statistics are presented in Table 2.

**Table 1:** Description of the variables.

**Types**	**Variables**	**Symbols**	**Definitions**
Dependent variables	Inpatient expenditure	*In_exp*	The logarithm of the per capita expenditure in hospitalization
	Outpatient expenditure	*Out_exp*	The logarithm of the per capita expenditure in outpatient
Independent variables	EPU	*EPU*1	China's provincial EPU index was proposed by Yu et al. (28)
		*EPU*2	The standardization of China's provincial EPU index was proposed by Yu et al. (28)
Control variables	GDP	*GDP*	The logarithm of GDP
	Aging rate	*Aging_rate*	The ratio of people over 65 to the total population
	Urbanization level	*Urban_rate*	The ratio of the urban population to the total population
	Industrial structure	*Indu_rate*	The proportion of the third industry in the economic structure
	Mortality rate	*Mor_rate*	The ratio of the deaths to the total population
	Number of medical institutions	*Hos_num*	The logarithm of the total number of medical institutions
	Fiscal revenue	*Fiscal_rev*	The logarithm of the total number of government fiscal revenue
	Number of beds in medical institutions	*Bed_num*	The logarithm of the total number of beds in medical institutions per 10,000 people

**Table 2:** Descriptive statistics of all variables.

**Variables**	**Obs**	**Mean**	**S.D**.	**Min**	**Max**
*In_exp*	319	72.750	31.410	29.290	217.400
*Out_exp*	319	1.676	0.745	0.010	4.602
*EPU*1	319	4.444	0.478	1.411	6.051
*EPU*2	319	21.220	14.760	1.010	86.250
*GDP*	319	9.537	0.876	6.681	11.400
*Aging_rate*	319	9.688	1.905	5.473	14.410
*Urban_rate*	319	54.190	13.670	28.240	89.600
*Indu_rate*	319	42.970	9.279	28.300	80.560
*Mor_rate*	319	5.737	1.103	2.280	7.400
*Hos_num*	319	2.759	0.720	0.470	4.069
*Fiscal_rev*	319	6.935	0.966	3.768	9.091
*Bed_num*	319	9.932	0.896	7.333	11.310

### Methods

#### Spatial Autocorrelation Test

Exploratory spatial data analysis (ESDA) is usually used to carry out the spatial autocorrelation test (39). Hence, we adopt the global and the local Moran index in ESDA to test the spatial correlation. The calculation formulas are as follows:

(1) $$\begin{array}{r} I_{global}=\frac{\overset{n}{\underset{i=1}{\sum }}\overset{n}{\underset{j=1}{\sum }}w_{ij}\left(x_{i}-\bar{x}\right)\left(x_{j}-\bar{x}\right)}{S^{2}\overset{n}{\underset{i=1}{\sum }}\overset{n}{\underset{j=1}{\sum }}w_{ij}} \end{array}$$

(2) $$\begin{array}{r} I_{local}=\frac{\left(x_{i}-\bar{x}\right)}{S^{2}}\overset{n}{\underset{j=1}{\sum }}w_{ij}\left(x_{j}-\bar{x}\right) \end{array}$$

where, $\bar{x}=\frac{1}{n}\overset{n}{\underset{i=1}{\sum }}x_{i}$, $S^{2}=\frac{1}{n}\overset{n}{\underset{i=1}{\sum }}{\left(x_{i}-\bar{x}\right)}^{2}$; *I*_*global*_ and *I*_*local*_ represent the global Moran index and local Moran index, respectively; *x*_*i*_ and *x*_*j*_ are the observed variables of region *i* and region *j*, respectively; $\bar{x}$ is the mean value of the related variable; *S*^2^ is the variance of the related variable; *n* is the total number of regions; *w*_*ij*_is the element of the spatial weight matrix.

Furthermore, it is essential to construct spatial weighting matrices to describe the relationship between different regions. In this study, referring to the study of Zeng et al. (27), we construct three spatial weight matrices (including spatial contiguity weight matrix, spatial distance weight matrix, and spatial economic weight matrix) to examine the spatial effects of EPU on healthcare expenditure.

The spatial contiguity weight matrix (W1) is constructed as follows:

(3) $$\begin{array}{r} w_{ij}=\left\{ \begin{array}{c} 1,\;i\neq j\\ 0,\;i=j \end{array}\right.\;i,j=1,2,\;\ldots ,\;n \end{array}$$

Besides, we construct the spatial distance weight matrix W2 to conduct the empirical analysis. The spatial distance weight matrix W_2_ is constructed as follows:

(4) $$\begin{array}{r} \;w_{ij}=\left\{ \begin{array}{c} 1/d_{ij},\;i\neq j\\ 0,\;i=j \end{array}\right.\;i,j=1,2,\;\ldots ,\;n \end{array}$$

Where, *d*_*ij*_ is the road distance between region *i* and region *j*.

However, the above two spatial weight matrixes only reflect the geographical relationship between regions, but they cannot reflect the influence of other factors (27). Hence, we also construct the spatial economic weight matrix W3 as follows:

(5) $$\begin{array}{r} W_{3}=W_{1}*\frac{1}{\bar{Y}}diag\left(\bar{Y_{1}},\bar{Y_{2}},\text{ L},\;\bar{Y_{n}}\right),\;i,j=1,2,\;\ldots ,n \end{array}$$

Where $\bar{Y_{i}}=\frac{1}{t_{1}-t_{0}+1}\overset{t_{1}}{\underset{t=t_{0}}{\sum }}Y_{it},\;Y=\frac{1}{n}\overset{n}{\underset{i=1}{\sum }}\bar{Y_{i}}$; *Y*_*it*_ is the per capita real GDP of the region *i* at year *t*; $\bar{Y_{i}}$ is the average per capita real GDP of the region *i* over the years; *Y* is the average per capita real GDP in all regions over the years.

#### Models

Prior literature mainly uses the three spatial econometric models, such as the spatial autoregression model (SAR), spatial errors model (SEM), and spatial Durbin model (SDM), to investigate the spatial effects between the related variables (40). Among them, SAR only contains the lagged term of dependent variables, SEM only contains the spatial spillover effects of independent variables, while SDM contains both the lagged term of dependent variables and the spatial spillover effects of independent variables. The above three spatial econometric models are defined as follows:

(6) $$\begin{array}{r} Y_{it}=\alpha +\rho W*Y_{it}+\alpha_{1}EPU_{it}+\beta {Control}_{it}+\sigma W*X_{kit}\\ \;+\mu_{it}\\ u_{it}=\lambda {Wu}_{it}+\varepsilon_{it},\varepsilon \sim N\left(0,\sigma^{2}I_{n}\right) \end{array}$$

Where *Y*_*it*_ represents the dependent variable of health expenditure; *EPU*_*it*_ represents the independent variable of EPU; *Control*_*it*_ represent the control variables, including *GDP, Aging_rate, Urban_rate, Indu_rate, Mor_rate, Hos_num, Fiscal_rev*, and *Bed_num*, respectively; *X* includes independent variable and control variables; **W** represents the spatial weight matrix; α represents the intercept item; α_1_ represents the coefficient of independent variable; β represents the coefficient of control variables; ρ represents the spatial autoregressive coefficient; λ represents the spatial error coefficient; θ represents the spatial lag coefficient. When ρ≠0 and λ = θ = 0, the model is SAR; λ≠0 and ρ = θ = 0, the model is SEM; when ρ≠0, θ≠0, and λ = 0, the model is SDM.

#### Model Selection

Consistent with the prior literature, we also use the Lagrange multiplier tests (i.e., LM-lag and LM-err) to select the spatial econometric model (41). The results are given in Table 3. All the results (including LM lag test, robust LM lag test, LM error test, and robust LM error test) are all significant at the 5% level, thus we should reject the null hypothesis of “no spatial autocorrelation.” Besides, the results of the Wald test and LR test are all significant at the 1% level. The above results indicate that we should apply the spatial econometric models to investigate the impact of EPU on healthcare expenditure in China. Because SDM includes both the lagged term of dependent variables and the spatial spillover effects of independent variables, we should select SDM for empirical analysis.

**Table 3:** Model selection test.

**Tests**	***t*-statistics**	***p*-values**
LM (lag) test	9.680	0.002
Robust LM (lag) test	5.683	0.017
LM (error) test	10.044	0.002
Robust LM (error) test	6.046	0.014
Wald test spatial lag	85.136	0.000
LR test spatial lag	37.666	0.000
Wald test spatial error	85.175	0.000
LR test spatial error	37.702	0.000

## Spatial Autocorrelation Analysis

We apply the Moran index to perform the spatial autocorrelation analysis. When the value of the Moran index is greater than zero, it indicates that there is a positive spatial correlation between data. On the contrary, when the value of the Moran index is smaller than zero, it indicates that there is a negative spatial correlation between data. The values of the global Moran index for the main variables are reported in Table 4. The results show that all the Moran index values of *In_exp* and *Out_exp* are positive and significant at the 5% level based on the two spatial matrices. These indicate that healthcare expenditure has obvious spatial autocorrelation in different regions of China. For EPU, some of their Moran index values are significant at the 10% level, which suggests that EPU of different regions is also spatial correlated with other regions.

**Table 4:** Global moran index values of healthcare expenditure and EPU.

**Year**	**Spatial contiguity weight matrix W1**				**Spatial economic weight matrix W3**			
	***In_exp***	***Out_exp***	***EPU*1**	***EPU*2**	***In_exp***	***Out_exp***	***EPU*1**	***EPU*2**
2007	0.266***	0.224***	0.073	0.207**	0.360***	0.284***	0.113**	0.138**
2008	0.280***	0.235***	−0.033	0.115*	0.373***	0.29***	−0.123*	−0.028
2009	0.274***	0.234***	0.021	−0.003	0.369***	0.287***	0.053*	0.035
2010	0.271***	0.228***	−0.065	−0.163	0.379***	0.316***	−0.062	−0.009
2011	0.259***	0.202***	−0.018	0.035	0.370***	0.283***	0.040*	0.115**
2012	0.251***	0.188**	0.001	0.123*	0.360***	0.252***	0.05	0.127**
2013	0.252***	0.175**	0.115*	0.178**	0.359***	0.239***	0.108**	0.105*
2014	0.258***	0.149***	−0.101	−0.031	0.362***	0.215***	−0.033	0.000
2015	0.263***	0.151**	−0.076	0.008	0.363***	0.227***	−0.045	0.037
2016	0.260***	0.143**	0.221***	−0.079	0.363***	0.228***	−0.059	−0.051
2017	0.266***	0.207**	−0.089	−0.062	0.360***	0.210***	−0.083	−0.114

***p < 1%, **p < 5%, and *p < 10%.

**Figure 3:**
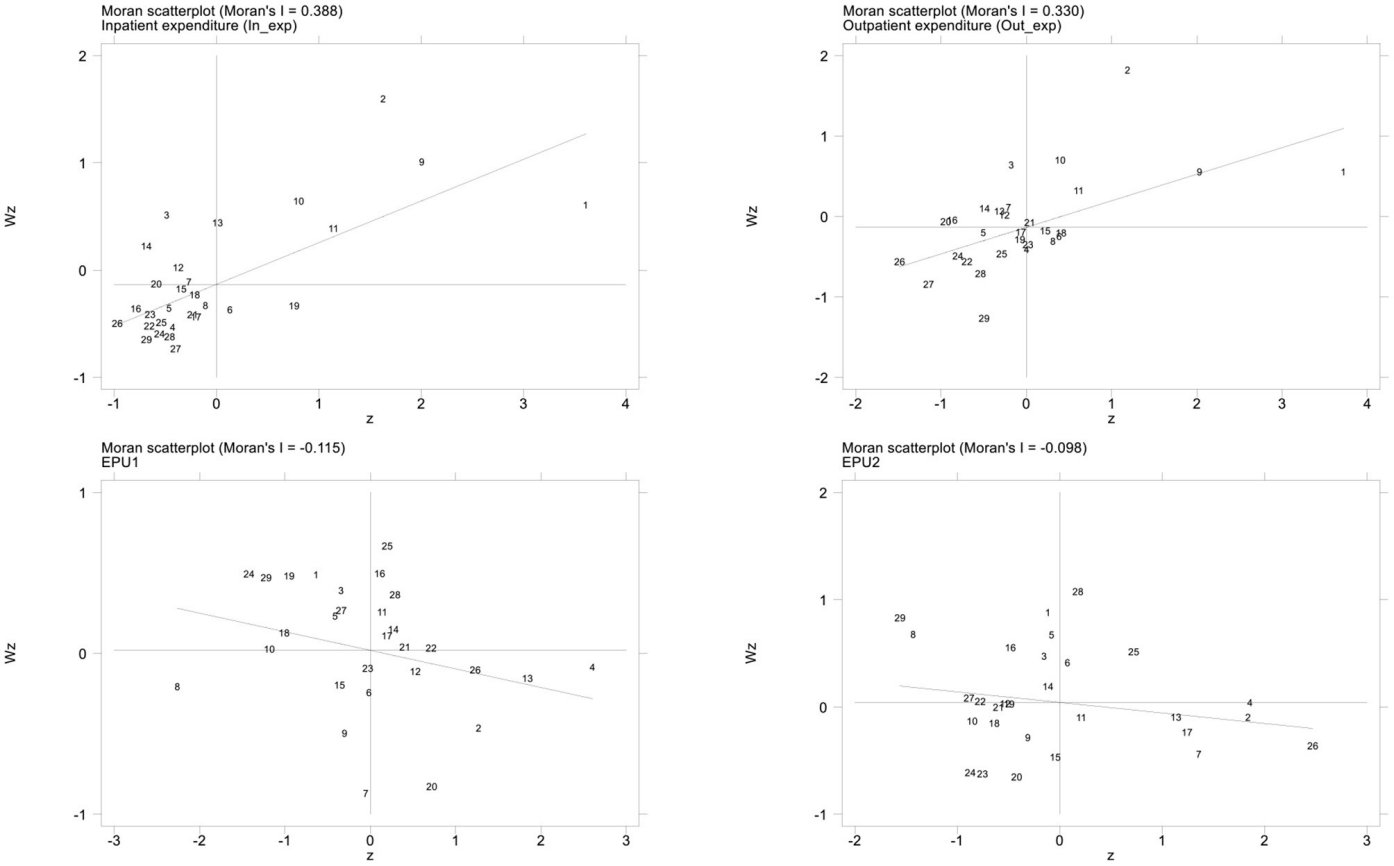
Scatter plot of the local Moran index values for healthcare expenditure and EPU (2017). Horizontal axis represents the observations of the local region, and the vertical axis represents the observations of the adjacent regions; The slope of the regression line in the scatter plot is equal to the global Moran index value.

We also use the local Moran index to examine the local agglomeration characteristics of healthcare expenditure and EPU. In the scatter plots of the local Moran index, the first quadrant and the third quadrant represent high-high (H-H) value clustering and low-low (L-L) value clustering, respectively. The second quadrant and the fourth quadrant represent low-high (L-H) value clustering and high-low (H-L) value clustering, respectively. Figure 3 gives the scatter plots of Moran index value using the spatial contiguity matrix W1 in 2017. We can find that, for healthcare expenditure (*In_exp* and *Out_exp*), most regions are located in the first quadrant and the third quadrant. These results suggest that healthcare expenditure has a positive spatial correlation with different regions. However, for the Moran index values of EPU (*EPU*1 and *EPU*2), most regions are located in the second quadrant and the fourth quadrant, which indicates that EPU has a negative spatial correlation in the different regions. These results are consistent with the global Moran index values in the above analysis. Therefore, it is necessary to consider spatial correlation when studying the impact of EPU on healthcare expenditure in China. Otherwise, the results may be biased.

## Empirical Analysis and Discussion

### Baseline Regression

We carry out the empirical analysis based on three spatial weight matrices (including spatial contiguity weight matrix W1, spatial distance weight matrix W2, and spatial economic weight matrix W3). Table 5 reports the estimation results using the SDM method. Among them, Column (1) is the estimation results based on the spatial contiguity weight matrix W1, Column (2) is the estimation results based on the spatial distance weight matrix W2, and Column (3) is the estimation results based on the spatial contiguity weight matrix W3.

**Table 5:** Estimation results of the impact of EPU on healthcare expenditure.

**Variables**	**Contiguity weight matrix W1**	**Distance weight matrix W2**	**Economic weight matrix W3**
	**(1)**	**(2)**	**(3)**
*EPU*1	0.9747***	1.1836***	1.1370***
	(3.16)	(3.83)	(3.11)
*GDP*	10.9685	16.4508**	7.4569*
	(1.21)	(2.08)	(1.73)
*Aging_rate*	−0.0720	−0.3310	0.5088
	(−0.20)	(−0.96)	(1.54)
*Urban_rate*	−1.6444***	−1.5419***	−0.8205***
	(−3.58)	(−2.98)	(−2.62)
*Indu_rate*	0.3598	0.3707	0.4048**
	(1.51)	(1.61)	(2.16)
*Death_rate*	1.5551	0.3055	−0.3009
	(1.52)	(0.29)	(−0.34)
*Hos_num*	−24.8311***	−29.6151***	−29.4146***
	(−2.64)	(−2.96)	(−3.95)
*Fiscal_rev*	8.6466	8.6837	14.0025***
	(1.60)	(1.55)	(2.97)
*Bed_rate*	1.0982	0.0268	0.9478
	(0.54)	(0.01)	(0.42)
*_cons*	−2.8e + 02***	−3.0e + 02***	−3.6e + 02***
	(−5.61)	(−5.12)	(−5.86)
W*EPU1	−0.6815	5.4321***	1.8449*
	(−1.04)	(3.18)	(1.85)
W*GDP	30.8799**	11.5531	45.0768***
	(2.32)	(0.93)	(4.02)
W*Aging_rate	0.4442	2.5889**	1.2100
	(0.71)	(2.03)	(1.05)
W*Urban_rate	1.2365**	2.2339	−1.4553**
	(1.97)	(1.47)	(−2.46)
W*Indu_rate	1.0346***	1.7396***	1.7709***
	(4.09)	(3.52)	(4.77)
W*Death_rate	−1.5752	−0.0983	0.6859
	(−1.63)	(−0.10)	(0.82)
W*Hos_num	−21.8530*	−70.4759**	−51.3869***
	(−1.80)	(−2.22)	(−2.68)
W*Fiscal_rev	−6.9207	15.1669	0.0631
	(−0.87)	(1.61)	(0.01)
W*Bed_rate	−0.2737	1.4840	0.7197
	(−0.13)	(0.70)	(0.34)
ρ	0.3945***	0.3011*	0.5840***
	(3.96)	(1.95)	(11.71)
θ	−3.1937***	−3.3012***	−2.7335***
	(−8.31)	(−7.94)	(−4.82)
*sigma2_e*	11.5810***	11.7754***	10.0067***
	(8.73)	(8.25)	(9.32)
Year FE	Yes	Yes	Yes
Region FE	Yes	Yes	Yes
*N*	319	319	319

This table presents the regression results about the impact of EPU on healthcare expenditure using SDM method with time and entity fixed effects; ***p < 1%, **p < 5%, and *p < 10%, respectively; t–statistics value in brackets.

The spatial autoregressive coefficients ρ are all positive and significant at the 10% level based on the three spatial weight matrices. These results indicate that there are spatial spillover effects of healthcare expenditure between a given region and geographically or economically connected regions. In other words, healthcare expenditure is influenced not only by factors such as EPU in a given region but also by healthcare expenditure in neighboring or economically connected regions. These conclusions are consistent with the findings of Zeng et al. (27). For the spatial lag coefficients θ, all the coefficients are significant at the 1% level, indicating that factors such as the EPU can affect healthcare expenditure not only in the given region itself but also in other nearby or economically connected regions. The coefficients of *sigma2_e* are all also significant at the 5% level. All these results suggest that there are spatial spillover effects between EPU and healthcare expenditure in China. Therefore, it is indispensable to introduce the spatial spillover effects when exploring the impact of EPU on healthcare expenditure. Otherwise, the conclusions will be biased.

The coefficients of EPU (*EPU*1) are all significant and positive at the 1% level (the coefficients are 0.9747, 1.1836, and 1.1370, respectively; the corresponding *t-*values are 3.16, 3.83, and 3.11, respectively) when we apply for the three spatial weight matrices. It indicates that healthcare expenditure in a particular region is positively correlated with the EPU of the region. The possible reason is that with the increase of EPU, residents face greater psychological and life pressure, which increases the possibility of seeking medical treatment. More important, when EPU increases, the government may exert more expenditure on health, such as the COVID-19 outbreak in 2019. Furthermore, the coefficients of the spatial lag of EPU (W^*^EPU1) are positive and significant at the 1% level and the 10% level, respectively, when we use the spatial distance weight matrix W2 and spatial economic weight matrix W3. However, the coefficient is insignificant at the traditional statistical levels based on the spatial contiguity weight matrix W1. These results indicate that the increase of EPU in a given region has a positive impact on healthcare expenditure in geographically close or economically connected regions. We also find that the spatial effects of EPU on healthcare expenditure are more influenced by geographically close and economically connected regions.

For all the control variables, the direct coefficients (*GDP*) and the spatial lag coefficients (W^*^GDP) of economic development are significantly positive at the traditional statistical levels, suggesting economic development in a region can increase the healthcare expenditure in a particular region or geographically close regions. The direct coefficients of urbanization level (*Urban_rate*) are negative and significant at the 1% level, indicating that urbanization is negatively related to healthcare expenditure in this region. The coefficients of the number of medical institutions (*Hos_num* and W^*^Hos_num) are all significantly negative at the 1% level, suggesting that the increase in the number of medical institutions in the local or geographical close regions will reduce the healthcare expenditure. The possible reason is that an increase in the number of medical institutions would promote the degree of competition, and reduce the medical price, which in turn would lead to a reduction in healthcare expenditure.

### Decomposition Effects of EPU on Healthcare Expenditure

The above conclusions show that there are significant spatial spillover effects in China's healthcare expenditure. Since SDM includes the spatial lag of dependent variables and independent variables at the same time, Lesage and Pace (42) decompose the total marginal effects into direct effects and indirect effects, which can better capture and explain the marginal effects of independent variables in SDM. Therefore, we further decompose the effects of EPU on healthcare expenditure (*In_exp*) into direct and indirect effects based on the spatial economic weight matrix W3, and the results are reported in Table 6.

**Table 6:** Decomposition effects of EPU on healthcare expenditure.

**Variables**	**Dependent variable:** ***In_exp***		
	**Direct effects**	**Indirect effects**	**Total effects**
*EPU*1	1.5158***	5.8162**	7.3321**
	(3.46)	(2.21)	(2.53)
*GDP*	14.4185***	113.0735***	127.4920***
	(3.33)	(3.95)	(4.20)
*Aging_rate*	0.7419**	3.1982	3.9401
	(2.32)	(1.23)	(1.46)
*Urban_rate*	−1.1047***	−4.4539***	−5.5586***
	(−3.16)	(−2.99)	(−3.28)
*Indu_rate*	0.6853***	4.5839***	5.2692***
	(3.63)	(4.57)	(4.88)
*Death_rate*	−0.1546	1.1334	0.9788**
	(−0.18)	(1.45)	(2.14)
*Hos_num*	−39.0581***	−1.5e + 02***	−1.9e + 02***
	(−3.81)	(−2.64)	(−2.87)
*Fiscal_rev*	15.0720***	17.7925	32.8645*
	(3.28)	(1.15)	(1.84)
*Bed_rate*	1.3537	2.7435	4.0973***
	(0.64)	(1.32)	(2.76)

***p < 1%, **p < 5%, and *p < 10%, respectively; t-statistics value in brackets.

It can be seen from the results in Table 6 that the coefficients of direct effects and indirect effects of EPU are 1.5158 and 5.8162, respectively, and they are significant and positive at the 5% level. These results indicate that EPU not only promotes the local healthcare expenditure but also has positive spatial spillover effects on the healthcare expenditure in the spatially related regions. The economic development (*GDP*) and the industrial structure (*Indu_rate*) can promote healthcare expenditure in the local region and also has significant positive spatial spillover effects on healthcare expenditure of the economically connected regions. Urbanization level (*Urban_rate*) and the number of medical institutions (*Hos_num*) have negative spatial spillover effects on healthcare expenditure in economically connected regions. These findings further verify that EPU has spatial spillover effects on healthcare expenditure. It also indicates that using spatial econometric models to analyze the relationship between EPU and healthcare expenditure can avoid overestimating the direct effects and underestimating the spatial spillover effects.

### Furth Analysis: Regional Heterogeneity

There are obvious differences between the marketization process in the eastern region and that in the central and western areas of China. Compared with the eastern region with a higher level of economic development and more perfect medical conditions, the central and western areas are relatively backward in economic and medical conditions, and their health investment and medical level are relatively low. Thus, with the dramatic changes of EPU, there may be significant differences in healthcare expenditure among the three regions. Therefore, we further investigate the spatial spillover effects of EPU on healthcare expenditure in the eastern, central, and western areas.

Following the scope defined in the Regional Development Plan approved by the state in China, we divide our samples into three areas: the eastern area contains 10 regions: Beijing, Tianjin, Hebei, Liaoning, Shanghai, Jiangsu, Zhejiang, Fujian, Shandong, and Guangdong. The central area includes nine regions: Heilongjiang, Jilin, Shanxi, Inner Mongolia, Anhui, Jiangxi, Henan, Hubei, and Hunan. The western area consists of 10 regions: Guangxi, Chongqing, Sichuan, Guizhou, Yunnan, Shaanxi, Gansu, Qinghai, Ningxia, and Xinjiang. We conduct the regression estimation of regional heterogeneity using the SDM method, and the results based on the spatial economic weight matrix W3 are reported in Table 7. We can find that all the spatial autoregressive coefficients ρ in the eastern and central areas are significantly positive at the 1% level (their coefficients are 0.1196 and 0.1202, respectively; the corresponding *t*-values are 3.60 and 2.87, respectively). In contrast, the spatial autoregressive coefficient ρ is negative and significant at the 5% level in the west area. These results suggest that, to a certain extent, the spatial spillover effects of healthcare expenditure in the eastern and central areas are opposite to that in the western area. That is, healthcare expenditure in local regions has positive spatial spillover effects on geographically or economically similar regions in the eastern and central areas, while it has a negative spatial spillover effect in the western area. Besides, all the coefficients θ and *sigma2_e* are also significant at the 1% level, which further implies that there are spatial spillover effects of EPU on healthcare expenditure in the three areas.

**Table 7:** Estimation results of regional heterogeneity.

**Variables**	**Eastern area**	**Central area**	**Western area**
	**(1)**	**(2)**	**(3)**
*EPU*1	0.0344***	0.0451*	−0.0571
	(3.35)	(1.71)	(−1.22)
W*EPU1	0.0238***	0.0236	0.0762
	(2.62)	(1.56)	(1.03)
Control variables	Yes	Yes	Yes
ρ	0.1196***	0.1202***	−0.2151**
	(3.60)	(2.87)	(−2.46)
θ	−4.4107***	−1.6911***	−3.1585***
	(−12.16)	(−4.09)	(−9.10)
*sigma2_e*	9.0678***	5.3169***	5.1230***
	(9.28)	(3.42)	(3.84)
Year FE	Yes	Yes	Yes
Region FE	Yes	Yes	Yes
*N*	110	99	110

This table presents the regression results of the SDM method based on spatial economic weight matrix; The results of control variables are consistent with those in Table 5, and we do not report them in this table due to space limitations; ***p < 1%, **p < 5%, and *p < 10%, respectively; t-statistics value in brackets.

In the eastern and central areas, the coefficients of EPU (*EPU*1) are significant and positive at the 1% level and the 10% level, respectively (the coefficients are 0.0344 and 0.0451, respectively; the corresponding *t-*values are 3.35 and 1.71, respectively). However, its coefficients are negative but insignificant at the traditional statistical levels in the western area (the coefficient is −0.0571, and the corresponding *t-*value is −1.22). The above results indicate that EPU has significant positive effects on healthcare expenditure in the eastern and central areas, while the effects are not significant in the western area. Next, focusing on the estimated coefficient of EPU (W^*^EPU1), it is positive and significant at the 1% level in the eastern area (the coefficient is 0.0238, and the corresponding *t-*value is 2.62), but it is insignificant at the traditional statistical levels in the central and western areas. It implies that, in the eastern area, the increasing of EPU not only affects healthcare expenditure in the local region but also affects healthcare expenditure in geographically close or economically connected regions. However, the effects do not exist in the central and western areas.

### Robustness Tests

#### Alternative Measure of Healthcare Expenditure

In the above analysis, we take inpatient expenditure (*in_exp*) as the proxy variable of healthcare expenditure. In this subsection, we adopt outpatient expenditure (*out_exp*) to conduct a robustness test for the above conclusions, and its definition is given in Section Variables. The results are reported in Columns (1–3) of Table 8. We can find that all the main coefficients (*EPU*1, W^*^EPU1, ρ, θ, *sigma2_e*) are consistent with the previous results. These empirical results further suggest that our empirical results remain stable when using different proxy variables.

**Table 8:** Estimation results of robustness tests.

**Variables**	**Alternative measure of healthcare expenditure:** ***Out_exp***			**Alternative measure of EPU:** ***EPU*****2**			**GMM**
	**W1**	**W2**	**W3**	**W1**	**W2**	**W3**	
	**(1)**	**(2)**	**(3)**	**(4)**	**(5)**	**(6)**	**(7)**
*EPU*1	0.0387**	0.0347*	0.0305*				
	(2.18)	(1.87)	(1.80)				
*EPU*2				0.0351**	0.0424***	0.0458***	
				(2.52)	(2.72)	(2.86)	
W*EPU1	0.0301*	0.0658*	0.0346**				4.4436***
	(1.90)	(1.87)	(1.73)				(166.18)
W*EPU2				−0.0091	0.1774***	0.0540**	
				(−0.39)	(2.78)	(2.39)	
*_cons*	0.5169	1.2740	3.5758***	−2.8e+02***	−2.8e+02***	−3.4e+02***	
	(0.38)	(0.67)	(3.58)	(−5.62)	(−4.65)	(−5.83)	
Control variables	Yes	Yes	Yes	Yes	Yes	Yes	Yes
ρ	0.9444***	0.9444***	0.9563***	0.3904***	0.3234**	0.5878***	
	(84.78)	(87.32)	(123.51)	(3.87)	(2.15)	(11.94)	
θ	−1.3320***	−1.3464***	−1.7160***	−3.1956***	−3.3007***	−2.7442***	
	(−3.49)	(−3.76)	(−5.92)	(−8.38)	(−7.94)	(−4.77)	
*sigma2_e*	0.0187**	0.0189*	0.0167*	11.6378***	11.8403***	10.0025***	
	(2.02)	(1.85)	(1.82)	(8.96)	(8.36)	(9.62)	
Year FE	Yes	Yes	Yes	Yes	Yes	Yes	
Region FE	Yes	Yes	Yes	Yes	Yes	Yes	
*N*	319	319	319	319	319	319	319

This table presents the robustness test results; Columns (1–3) are the results of the alternative measure of healthcare expenditure (Out_exp) based on the three spatial weight matrices, respectively; Columns (4–6) are the results of the alternative measure of EPU (EPU2) based on the three spatial weight matrices; Column (7) is the result of the endogenous test using the GMM method; The results of control variables are consistent with those in Table 5, and we do not report them in this table due to space limitations; ***p < 1%, **p < 5%, and *p < 10%, respectively; t-statistics value in brackets.

#### Alternative Measure of EPU

In the above empirical tests, we use the EPU index proposed by Yu et al. (28) to measure the EPU. In this subsection, we adopt the standardization of China's provincial EPU index (*EPU*2) proposed by Yu et al. (28) to carry out the robustness test, and the definition of *EPU*2 is given in Section Variables. The regression results are reported in Columns (4–6) of Table 8 and show that all the coefficients are significant at the traditional statistical levels. These results suggest that our previous conclusions are robust, and further confirm that EPU has spatial spillover effects on healthcare expenditure in China.

#### Endogenous Test

In general, it is difficult for healthcare expenditure to affect EPU at the macro level. Thus, there is almost no inverse causality between EPU and healthcare expenditure. Besides, we control some related variables influencing healthcare expenditure in the previous empirical analyses, but it is impossible to avoid endogenous problems. Therefore, referring to the study of Elhorst (40), we use the spatial Generalized Method of Moments (GMM) to perform the endogenous test. Following Zeng et al. (27), we adopt W^*^EPU1 as the instrumental variable of the spatial GMM method. The results of the spatial GMM method are reported in Column (7) of Table 8. The coefficient of W^*^EPU1 is significant and positive at the 1% level, which further verifies the results of the spatial GMM estimation are consistent with the previous conclusions.

## Conclusions and Implications

This study aims to explore the impact of EPU on healthcare expenditure using the panel data of 29 Chinese regions from 2007 to 2017. Moreover, we also analyze the regional heterogeneity of the spatial effects between EPU and healthcare expenditure. For these purposes, we adopt the spatial Durbin model (SDM) to conduct empirical analyses based on the three spatial weight matrices.

This study is the first substantial research of the spatial spillover effects of EPU on healthcare expenditure in health economic studies. Our empirical results show that healthcare expenditure is not randomly distributed between regions in China, and has significant characteristics of spatial clustering and spatial spillover effects. In other words, local healthcare expenditure can exert positive spatial spillover effects on geographically or economically connected regions. We also find a positive correlation between EPU and healthcare expenditure. That is to say, EPU can affect healthcare expenditure not only in the local region itself but also in other geographically or economically connected regions. Moreover, our study further reveals that the above spatial spillover effects are heterogeneous in the eastern, central, and western areas. The spatial spillover effects of EPU on healthcare expenditure only exist in the eastern area, but not in the central and western areas.

Overall, our empirical findings highlight the spatial spillover effects of EPU on healthcare expenditure in emerging markets. However, there are some limitations in our study and further research is needed. First, the dataset of our study is limited to provincial data in China, which may limit the generality of the results. Future research can extend the dataset to the municipal level or even the county level to examine the spatial effects of EPU on healthcare expenditure. Second, due to the availability of data, our research only uses the three spatial weight matrices, further research can be extended to other weight matrices, such as the human resources weight matrix and public service weight matrix. Finally, one crucial future research is to investigate whether the COVID-19 pandemic affects the impact of EPU on healthcare expenditure because the COVID-19 pandemic led to unprecedented policy responses, such as lockdowns and stimulus packages (23, 43, 44).

## Data Availability Statement

The original contributions presented in the study are included in the article/supplementary material, further inquiries can be directed to the corresponding author/s.

## Author Contributions

PB: conceptualization, writing—original draft, and software. YT: data collection, literature search, and writing—original draft. WZ: writing—reviewing and editing and supervision. MZ: data collection, literature search. All authors contributed to the article and approved the submitted version.

## Conflict of Interest

The authors declare that the research was conducted in the absence of any commercial or financial relationships that could be construed as a potential conflict of interest.

## Footnotes

^1^Data source: https://data.stats.gov.cn.
